# Health-Related Quality of Life of People Living with Hansen’s Disease Undergoing Multidrug Therapy

**DOI:** 10.3390/tropicalmed11090254

**Published:** 2026-09-09

**Authors:** Victor Feitosa de Freitas, Liliane Lins-Kusterer, Karen Valadares Trippo, Herman Henrique Silva Santana, Fernando Martins Carvalho

**Affiliations:** 1 Graduate Program in Medicine and Health, Federal University of Bahia, Salvador 40110-909, BR, Brazil; victorterapia@hotmail.com (V.F.d.F.); lkusterer@gmail.com (L.L.-K.); hermansilvasantana@gmail.com (H.H.S.S.); 2 Graduate Program in Rehabilitation Sciences, Health Sciences Institute, Federal University of Bahia, Salvador 40110-909, BR, Brazil; ktrippo@ufba.br

**Keywords:** leprosy, drug polytherapy, quality of life, pain

## Abstract

Background: Multidrug therapy (MDT) is the cornerstone of treatment for Hansen’s disease. We aimed to identify factors associated with health-related quality of life (HRQoL) of people with Hansen’s disease undergoing MDT. Methods: Sixty-six people of any sex, aged 18 and older, undergoing MDT, without adverse reactions, were enrolled as outpatients at a public health service in Salvador, Brazil. A physical examination was performed, during which the Simplified Neurological Evaluation Form was used to determine the Degree of Physical Disability. The HRQoL was evaluated using the RAND-36 questionnaire and its Physical Component Summary (PCS) and Mental Component Summary (MCS). Results: The PCS and MCS were negatively associated with pain and positively associated with the Degree of Physical Disability after controlling for MDT duration, bacilloscopy, schooling, race/color, age, sex, and monthly income; the PCS was negatively associated with smoking. Patients who reported pain had a PCS 14.282 percent units lower than those who did not report it, and patients that reported pain had an MCS 6.762 units lower. Differences in such magnitude are clinically important for patients with Hansen’s disease being treated with MDT. Conclusion: Pain and the Degree of Physical Disability are strongly associated with the HRQoL of patients with Hansen’s disease undergoing MDT.

## 1. Introduction

Globally, 172,717 new cases of Hansen’s disease were identified in 2024. India reported 100,957 new cases; Brazil ranked second with 22,129 cases; and Indonesia ranked third with 14,698 cases. In 2024, approximately 10% of reported cases in Brazil were classified as having grade 2 physical disability, indicating late diagnosis, and 80% of cases were multibacillary, the most contagious form of the disease [1]. New cases of Hansen’s disease in Brazil predominantly occur among people with incomplete elementary education and illiterate individuals in the Northeast region [2]. These aspects are of extreme importance, as they are associated with these individuals’ quality of life.

Hansen’s disease is a contagious infectious disease that affects the peripheral nerves and the skin; if left untreated, it can cause pain, deformities, and physical disabilities that are sometimes irreversible and have significant social, emotional, and psychological impacts [3]. A key feature of the pathogenesis of Hansen’s disease is inflammation in and around neural tissue, which is driven, in part, by immune responses spanning the disease’s entire immunological spectrum. Non-specific inflammatory phenomena related to inflammation, infection, and foreign bodies may also arise, sometimes triggering and/or exacerbating painful processes and loss or a reduction in physical capacity [4]. The disabilities and deformities caused by leprosy extend beyond the physical realm, affecting patients’ quality of life, with obvious trouble in performing activities of daily living, a lack of interaction with the community, and psychological impacts [5]. Early diagnosis and prevention of the complications and sequelae of leprosy must begin at the primary care level, using multidrug therapy (MDT) as the standard treatment [6].

The repercussions of Hansen’s disease are not limited to clinical manifestations. Physical disabilities, functional limitations, and sensory changes can impair the performance of activities of daily living, return to work, and social participation, resulting in consequences that directly affect the quality of life of affected individuals [3]. Health-Related Quality of Life (HRQoL) is an important outcome in clinical research, as it reflects an individual’s perception of the effects of a disease on different aspects of their life. It is a multidimensional concept that encompasses physical, psychological, and social aspects and is widely assessed using standardized instruments, such as the RAND-36 [7] and the Short Form-36 (SF-36) [8], especially in studies conducted in countries in which leprosy is endemic [9,10,11]. Health-related quality of life for individuals with Hansen’s disease can be influenced by various clinical and psychosocial factors, including physical disabilities, relapse episodes, neuropathic pain, limitations in work, perceptions of social exclusion, and the use of MDT [12,13,14]. These factors may have distinct effects on the domains assessed by quality-of-life instruments, highlighting the multifactorial nature of leprosy’s impact. Despite the convergence of findings, the literature still has significant limitations: observational cross-sectional studies predominate, with a scarcity of longitudinal studies and differences in the standardization of participants’ clinical stratification.

This study was conducted to identify factors associated with health-related quality of life among people with leprosy undergoing multidrug therapy.

## 2. Methods

### 2.1. Study Design and Data Collection

This cross-sectional study investigated people with Hansen’s disease who were in treatment with MDT for six or 12 months for paucibacillary or multibacillary disease, respectively, and treated as outpatients at the Instituto Couto Maia, a public health service provider in Salvador, Brazil. From May to July, 2024, patients were invited to attend the Institute for a physical examination and an interview about their health-related quality of life. According to the Ministry of Health’s protocol, patients must attend monthly to take their supervised dose and are reevaluated every 3 months using the Simplified Neurological Assessment. The hospital’s social services department has an effective program for actively tracking patients undergoing treatment using mail, phone, social media, email, and home visits. None of the patients undergoing treatment failed to attend their appointments. Hansen’s disease is a condition associated with by many stigmas and a great deal of fear, particularly regarding leprosy-related reactions and physical sequelae. These factors could explain the high level of adherence to the program and treatment.

People of any sex aged 18 years or older were included; those presenting with reactions to MDT, such as erythema nodosum leprosum or reversal reaction, were excluded. The reasons for excluding these patients were as follows: 1. Minimizing confounding bias—Hansenic reactions (Type 1/Reverse and Type 2/Hansenic Erythema Nodosum) are acute, episodic inflammatory events. The severe pain, systemic symptoms (fever, malaise, edema, myalgia, arthralgia, fatigue), and functional impairment associated with these reactions represent a temporary and fluctuating state of exacerbation. Including them would introduce a strong confounding bias in the quality of life scores, making it more difficult to isolate the true impact of the underlying chronic condition. 2. Sample homogeneity—patients experiencing a reactive flare-up exhibit a clinical course that is radically different from that of patients in a stable chronic state. Mixing these two profiles would result in high data heterogeneity, compromising the internal validity of the study and the reproducibility of the results. 3. The use of high-dose corticosteroids and immunosuppressants—the treatment of reactive episodes requires aggressive therapeutic regimens (such as high doses of prednisone and/or thalidomide). These medications alone significantly alter the patient’s pain, functional capacity, mood, and perception of quality of life during the reaction and may act as a pharmacological confounding variable. 4. Temporal fluctuation in quality-of-life indicators—the questionnaire was administered based on the patient’s condition at a specific point in time. Measuring an individual’s quality of life during an acute crisis could yield an extremely low point-in-time value that does not reflect the disease’s impact on the individual, thereby distorting the study’s conclusions.

The patients signed an Informed Consent Form. Procedures were adopted to protect the secrecy and confidentiality of information obtained from each patient. The project was evaluated and approved by the Ethics Committee of the School of Medicine of Bahia.

### 2.2. Variables and Measurement Instruments

The dependent variable was health-related quality of life, evaluated using the RAND-36 (RAND 36-Item Health Survey) questionnaire, which is composed of 36 items that encompass eight domains: Role Physical (RP), Bodily Pain (BP), General Health (GH), Vitality (VT), Social Functioning (SF), Role Emotional (RE), and Mental Health (MH). These domains can be used to build a Physical Component Summary (PCS) and a Mental Component Summary (MCS) [7]. Raw domain scores and component summaries can range from 0 to 100. Raw scores were normalized with a mean of 50 and a standard deviation of 10, using normative data for the population of the United States of America, according to the RAND-36 questionnaire developers. Higher scores indicate better health-related quality of life. A normalized score below 50 is lower than the expected level for the population of the USA [15].

The RAND-36 and the SF-36 questionnaires have identical content, but the SF-36 is commercially available and more frequently used. Because they use different scoring algorithms, RAND-36 yields slightly different scores in the Bodily Pain and General Health domains. However, both the SF-36 and RAND-36 scores show a strong correlation coefficient (r = 0.99) for the Bodily Pain and General Health domains [16].

The independent variables were age, sex, race/color, schooling, monthly income, smoking, alcohol consumption, complaint of pain, disease severity as perceived by the patient, duration of multidrug therapy (measured in months), and Degree of Physical Disability. These pieces of information were collected using a semi-structured questionnaire specifically designed for this study.

The Degree of Physical Disability due to leprosy (DPD) was evaluated according to the directions for completing the Simplified Neurological Evaluation Form for patients with Hansen’s disease, recommended by the Brazilian Ministry of Health, following WHO directives [17], and classified as Grades 0, 1, or 2 [6]. The DPD form primarily focuses on evaluating structures and visible body functions, such as the eyes, hands, and feet. Muscle strength was evaluated using the Medical Research Council muscle strength scale, and skin sensibility using Semmes–Weinstein nylon monofilaments (SME, Inc., Wilmington, NC, USA). Importantly, the DPD form does not include an evaluation of pain. The Simplified Neurological Evaluation Form was administered by the first author of this article, who has experience in this field.

### 2.3. Statistical Analyses

Exploratory factor analysis was used to evaluated the adequacy of the RAND-36 in the studied population with JASP 0.17.3 [18]. Data adequacy for factor analysis included the Kaiser–Meyer–Olkin (KMO) test and Bartlett’s test of sphericity [19]. Data were processed and analyzed using the Statistical Package for the Social Sciences™—SPSS, version 20.0 (IBM Corp, Armonk, NY, USA).

The reliability of the RAND-36 questionnaire was evaluated by the Composite Reliability Index (CRI). Reference values are ≥0.70 good reliability [20].

#### Multiple Linear Regression Analyses

*T* tests for independent samples, one-way ANOVA, and Kruskal–Wallis ANOVA, assuming *p* < 0.20 as a criterion value, were used to select the independent variables that compose the two multiple linear regression equations that have the Physical and the Mental Component Summaries as dependent variables. The variable multidrug therapy was included in each equation, even though it did not meet the *p* < 0.20 criterion. The ENTER method was used to run the multiple linear regression models. Cases that varied by more than ±3 standard deviations in the studentized residual analysis were excluded from the corresponding equation. The residual analysis considered two types of scattergrams: (a) regression of standardized residuals, plotting the observed cumulative probability against the expected cumulative probability; and (b) regression of the studentized residuals against the observed values of PCs and MCS.

The multiple linear regression technique was used to adjust the raw and standardized coefficients (or BETA coefficients, obtained by transforming the data into Z-scores before running the regressions). BETA coefficients allow direct comparisons among independent variables to be made, because they use the same scale of measurement [21].

## 3. Results

The 66 people included in this study were outpatients undergoing multidrug therapy for Hansen’s disease for 4.2 months ± 3.7 (mean ± SD), 2.5 months (median); 1.0–12 months (range). Mean age was 50.7 years ± 15.9 (SD), and the median was 53.0 years. Raw scores of the RAND-36 domains and the normalized scores for the Physical and Mental Component Summaries are presented in Table 1. Medians of PCS and MCS were 49.4 and 43.6, respectively.

The suitability of the RAND-36 data for factor analysis was confirmed by the Kaiser–Meyer–Olkin measure (KMO = 0.821), indicating good sample adequacy, and by Bartlett’s sphericity test, which demonstrated significant correlations among the items (χ^2^ = 2447.700; df = 630; *p* < 0.001). These results supported the conduct of exploratory factor analysis. Regarding the eight RAND-36 domains, the Cronbach’s alphas ranged from 0.714 for General Health to 0.966 for Role Physical.

PCS was markedly lower among patients who were Black or Indigenous, had completed nine or fewer years of schooling, were smokers (Table 2), had multibacillary Hansen’s Disease, reported pain, or had a physical disability grade of 0 or 1. MCS was markedly lower among patients with lower monthly income, those who reported pain, and those with a physical disability grade of 0 or 1 (Table 3).

The pure neural form was found in 8 (12.2%) of the 66 patients (Table 4). Referred pain was reported by three (37.5%) of patients with pure neural form, and by 44.8% of those without this form of the disease.

Pain was most frequently (69.2%) reported by patients with Degree of Physical Disability 1 and less frequently (34.1%) reported by those with DPD 2 (Table 4).

Patients’ years of age showed a negative linear regression correlation with PCS (r = −0.029; *p* = 0.819) and a positive linear correlation (r = 0.27; *p* = 0.027) with MCS.

Multiple linear regression analyses revealed that the Physical Component Summary (Equation (1)) was associated with pain (*p* < 0.001). The model estimated that patients who reported pain had a PCS mean score 14.282 percent units lower than those who did not report it, confirming the 9.5 percent unit (47.0–37.5%) difference observed in the bivariate analysis. Estimated PCS scores were 8.119 lower for smokers and 6.609 higher for those with a Degree of Physical Disability 2. Compared with the other seven variables in the equation, pain had the highest Beta coefficient (−0.535). The Mental Component Summary was strongly associated with pain and with the Degree of Physical Disability 2. Equation (2) estimates that the mean Mental Component Summary score was 6.762 percent units lower among those who reported pain and 10.571 units higher among those with a higher degree of physical activity 2 compared to those with a Degree of Physical Disability 0. The multiple linear regression model was adequate for analyzing data from Equations (1) and (2), as indicated by ANOVA *p*-values of <0.001 and <0.002, respectively. Equations (1) and (2) presented satisfactory values (PCS = 0.575 and MCS = 0.339) for the determination coefficient R^2^, which indicates the proportion of the dependent variable explained by the independent variables in the model. Residual analysis revealed one outlier with a studentized residual of −3.260 in Equation (1). Excluding this outlier did not substantially change the results in the equation. Collinearity among the predictors did not provide important limitations to the analyses. A VIF below 3 indicates no multicollinearity. Variance Inflation Factor (VIF) was always low for Equation (1), ranging from 1.0919 to 1.931, as well as for Equation (2), ranging from 1.091 to 1.880. The Durbin–Watson statistic was 2.083 and 2.203 in Equations (1) and (2), respectively. The Durbin–Watson statistic detects autocorrelation in regression residuals. Values near 2.0 are ideal; values above 1.5 or below 2.5 are acceptable [21] (Table 5).

## 4. Discussion

This study yielded the following key findings: 1. The physical and mental components of the health-related quality of life of people with Hansen’s disease undergoing multidrug therapy were negatively associated with pain and positively associated with the Degree of Physical Disability. after controlling for other relevant variables; 2. further, the physical component was negatively associated with smoking.

In this population, the Pain variable is most strongly associated with the physical component of health-related quality of life. Patients who reported pain had a PCS 14.282 units (%) lower than those who did not report it. A difference of such magnitude is probably clinically important for patients with Hansen’s disease. Minimal Clinically Important Difference (MCID) represents the smallest change in a patient-reported outcome measure, such as RAND-36 and SF-36 scores, that the patient perceives as beneficial or meaningful [22]. To the best of our knowledge, the MCID for the Physical and Mental Component Summaries (PCS and MCS) has not yet been determined for people with Hansen’s disease. In patients with chronic pain, MCID estimates were 2.62 to 4.69 for PCS and 4.46 to 6.79 for MCS [23], and, in patients who underwent musculoskeletal oncologic surgery, 5.0 for both measures [24].

The tool used to classify the Degree of Physical Disability for leprosy (DPD), the Simplified Neurological Evaluation Form, does not include pain as an assessment domain. This gap is relevant because pain can profoundly compromise functionality and social participation, even in the absence of visible deformities or detectable sensory loss, leading to an underestimation of the patient’s actual disability. The absence of pain in DPD assessment can also hinder the identification of individuals who require specific management of this symptom, rehabilitation, or psychosocial support, in addition to introducing biases in epidemiological data and health service planning. From a care perspective, not including pain in the multidimensional instrument reduces its sensitivity for monitoring interventions and may perpetuate physical suffering and Mental Health impacts, which are frequently associated with neuropathic or inflammatory pain in leprosy. Incorporating pain assessment into the instrument for evaluating the Degree of Physical Disability could contribute to better guidance for clinical decisions, rehabilitation, and public policies regarding leprosy.

In this study, multivariate analysis estimated that patients who smoke have an 8.119- point lower score on the health-related quality of life physical component. Leprosy attacks the skin and peripheral nerves [17]; concurrently, smoking heavily exacerbates these effects by impairing skin elasticity, reducing the thickness and density of the dermis and epidermis [25], affecting peripheral circulation [26], and worsening neuropathic pain [27], thereby delaying the healing of leprosy-related ulcers and contributing to worse quality of life.

In this study, multivariate analysis revealed that patients with a physical disability degree of 2 reported higher quality of life scores in the mental domain, while pain was associated with lower scores. Bivariate analysis has shown that patients with DPD 2 reported the lowest frequency (34.1%) of pain compared to those with other Degrees of Physical Disability. These associations likely result from severe, permanent loss of protective sensation due to advanced destruction of peripheral nerve fibers by *Mycobacterium leprae*, as is characteristic of DPD 2 [28]. Consequently, this neurological impairment attenuates active nociceptive input, resulting in a significantly lower prevalence of reported pain in our Grade 2 cohort (34.1%) compared with Grade 1 patients (69.2%). Furthermore, the higher proportion (41/66 = 62.1%) of patients with DPD 2 suggests the occurrence of a high proportion of late diagnosis in this population.

To the best of our knowledge, no other study has used RAND-36 in patients with characteristics similar to our study that investigated outpatients with Hansen’s disease who were in treatment with MDT for paucibacillary or multibacillary disease, respectively, of any sex, aged 18 years and older, and without an adverse reaction to MDT, like erythema nodosum leprosum or reversal reaction. However, four studies with populations comparable to ours used the SF-36 [10,12,13,14].

Only one study from West Bengal, India, with 113 newly diagnosed patients with leprosy who were on MDT or had recently completed MDT, with no reactions or neuritis, used the SF-36 Physical Component Summary (71.2 ± 23.5) and Mental Component Summary (80.8 ± 21.8) [10]. However, these component scores were not normalized, hampering comparison with our results. The SF-36 developers strongly recommend score normalization [29].

In our study, the raw mean score of the Bodily Pain domain was 57.1 ± 38.9. This score is much lower than those reported by other studies, at 87.2 ± 18.9 [12], 75.2 ± 41.7 [10], 79.6 ± 9.7, and 73.9 ± 10.8 [13], but much higher than the 33.6 ± 21.5 and 24.2 ± 12.7 [14] reported by patients with leprosy-related neuropathic pain. In our study, only a few (eight) patients presented with the pure neural form of Hansen’s disease, among whom only three had referred pain. Therefore, pain related to the neurological form of the disease does not seem to play an important role in the lower scores of the physical and mental components of their health-related quality of life. Pain is an unpleasant sensory and emotional experience that is entirely subjective. The perception of pain, which is processed by the central nervous system, can vary widely depending on a person’s physiological, psychological, and cultural characteristics, as well as their past experiences.

Nerve decompression surgery resulted in PCS mean scores higher than in the rest of the study group, but only three patients underwent this procedure. This surgery aims to decompress and/or release a compromised peripheral nerve, usually the ulnar, median, radial, common peroneal, or posterior tibial nerve. This surgery is primarily indicated in cases of persistent or progressive leprosy-related compressive neuropathy, characterized by severe, refractory neuropathic pain or progressive functional impairment [30,31]. Nerve decompression surgery is not a routine procedure for all patients with nerve damage. Its indication must be determined on a case-by-case basis following a thorough surgical and dermatoneurological evaluation, and it is reserved for patients with a formal contraindication to corticosteroid use, nerve abscesses, patients with neuritis that does not respond to medical treatment within four weeks, recurrent or new leprosy-related reactions, patients with ulnar nerve subluxation, or when there is an imminent risk of irreversible physical disabilities and/or deformities [31].

This study has some limitations that warrant consideration. The cross-sectional design precludes definitive causal inference, and the sample size of 66 patients reflects a specific clinical population. Furthermore, the high prevalence of Grade 2 physical disability (62.1%) indicates a selection bias inherent to data collection at a tertiary reference center, which may limit the generalizability of the findings to primary healthcare settings. Nonetheless, these limitations are heavily countered by significant methodological strengths. A major advantage is that a single, highly experienced investigator conducted all clinical evaluations, completely eliminating inter-rater variability. Additionally, the stringent eligibility criteria, specifically the exclusion of patients with leprosy reactions, enabled a precise assessment of the baseline impact of multidrug therapy and chronic pain on quality of life. Finally, the RAND-36 survey demonstrated robust psychometric performance in this population and internal consistency, ensuring the reliability of the collected data.

We concluded that the health-related quality of life of this study population was associated with pain and the Degree of Physical Disability, and smoking was negatively associated with its physical component. The inclusion of the domain of pain in the Simplified Neurological Evaluation Form, the tool used to classify the Degree of Physical Disability, could provide better guidance for clinical decisions, rehabilitation, and public policies regarding leprosy.

## Figures and Tables

**Table 1:** RAND-36 scores of 66 individuals with Hansen’s disease undergoing. multidrug therapy, Salvador, Brazil, 2024.

Domain ^1^/Summary Component ^2^	Mean ± SD	Median
Physical Functioning (PF)	69.5 ± 34.7	90.0
Role Physical (RP)	63.8 ± 39.5	87.5
Bodily Pain (BP)	57.1 ± 38.9	52.5
General Health (GH)	68.7 ± 23.0	75.0
Vitality (VT)	55.7 ± 29.2	55.0
Social Functioning (SF)	68.8 ± 37.8	100.0
Role Emotional (RE)	53.5 ± 47.8	66.7
Mental Health (MH)	59.7 ± 26.2	62.0
Physical Component Summary (PCS)	46.0 ± 13.7	49.4
Mental Component Summary (MCS)	42.8 ± 14.5	43.6

^1^ Raw scores; ^2^ Normalized scores.

**Table 2:** RAND-36 component summary scores (mean ± SD) according to the characteristics of the 66 patients with Hansen’s disease undergoing multidrug therapy, Salvador, Brazil, 2024.

Characteristics	Strata	*n*	Physical Component Summary	*p* ^1^	Mental Component Summary	*p* ^1^
Sex				0.494		0.124
	Masculine	31	44.7 ± 13.2		45.7 ± 14.3	
	Feminine	35	47.1 ± 14.3		40.2 ± 14.4	
Race/Color				0.003		0.059
	White/Yellow	12	53.8 ± 7.8		49.9 ± 12.9	
	Black/Indian	54	44.2 ± 14.2		41.2 ± 14.5	
Schooling (years)				0.018		0.813
	9	26	41.0 ± 14.6		42.3 ± 16.1	
	≥10	40	49.1 ± 12.3		43.2 ± 13.6	
Monthly income (US$)				0.373		0.004
	<260	48	45.0 ± 13.9		39.7 ± 14.3	
	≥260	18	48.4 ± 13.4		51.0 ± 11.8	
Smoking				0.006		0.387
	Yes	9	35.9 ± 9.2		38.5 ± 14.4	
	No	59	47.5 ± 13.7		43.5 ± 14.5	
Drinking				0.206		0.930
	Yes	30	43.6 ± 12.7		42.6 ± 14.6	
	No	36	47.9 ± 14.5		43.0 ± 14.9	

^1^ Using *t*-test.

**Table 3:** RAND-36 component summary scores (mean ± SD) according to the clinical characteristics of 66 patients with Hansen’s disease undergoing multidrug therapy, Salvador, Brazil, 2024.

Clinical Characteristics	Strata	*n*	Physical Component Summary	*p*	Mental Component Summary	*p*
Pain				<0.001 ^1^		0.007 ^1^
	Yes	29	37.3 ± 11.7		37.5 ± 14.0	
	No	37	52.8 ± 11.2		47.0 ± 13.6	
Pure neural form				0.508 ^2^		0.150 ^2^
	Yes	8	42.2 ± 12.7		35.4 ± 16.6	
	No	60	45.7 ± 14.3		43.3 ± 14.2	
Nerve decompression surgery				0.493 ^2^		1.000 ^2^
	Yes	3	52.8 ± 7.0		42.2 ± 17.0	
	No	65	45.0 ± 14.2		42.4 ± 14.6	
Disease degree ^3^				0.473 ^4^		0.368 ^4^
	Mild	4	45.6 ± 12.7		48.5 ± 9.3	
	Moderate	14	49.3 ± 11.1		46.8 ± 12.2	
	Severe	48	45.0 ± 14.6		41.2 ± 15.3	
Degree of Physical Disability				0.004 ^5^		0.043 ^5^
	0	12	39.7 ± 13.3		34.3 ± 13.1	
	1	13	38.0 ± 9.3		40.4 ± 15.1	
	2	41	50.3 ± 13.4		46.0 ± 13.9	
Bacilloscopy				0.001 ^1^		0.834 ^1^
	Paucibacillary	10	56.4 ± 7.8		41.9 ± 12.3	
	Multibacillary	56	44.1 ± 13.8		43.0 ± 15.0	
MDT (months)				0.439 ^1^		0.648 ^1^
	0–6	48	46.8 ± 13.6		43.8 ± 14.2	
	7–12	18	42.3 ± 13.8		44.1 ± 16.6	

^1^ *t*-test; ^2^ Mann–Whitney test; ^3^ As perceived by the patient; ^4^ Kruskal–Wallis ANOVA; ^5^ One-Way ANOVA.

**Table 4:** Degree of Physical Disability (DPD) according to pain reported by 66 patients with Hansen’s disease undergoing multidrug therapy, Salvador, Brazil, 2024.

DPD	Yes		Pain No		Total	
	N	%	N	%	N	%
0	6	50.0	6	50.0	12	100.0
1	9	69.2	4	30.8	13	100.0
2	14	34.1	27	65.9	41	100.0
Total	29	43.9	37	56.1	66	100.0

**Table 5:** Multiple linear regression equations with the Physical Component Summary (PCS) and Mental Component Summary (MCS) in %, as dependent variables, among patients with Hansen’s disease undergoing multidrug therapy, Salvador, Brazil, 2024.

Predictor (Referent)	Equation (1) (Dependent = PCS) *n* = 65				Equation (2) (Dependent = MCS) *n* = 66			
	b ^1^	Std Error of b	*p*	Beta ^2^	b ^1^	Std Error of b	*p*	Beta ^2^
Multidrug therapy (0–6 months)	−2.433	2.700	0.371	−0.082	1.285	3.691	0.729	0.040
Bacilloscopy (Paucibacillary)	−4.036	3.578	0.264	−0.110				
Pain (No)	−14.282	2.531	<0.001	−0.535	−6.762	3.353	0.048	−0.233
Degree of Physical Disability 1 (0)	2.580	3.971	0.519	0.078	8.507	5.151	0.104	0.235
Degree of Physical Disability 2 (0)	6.609	3.304	0.050	0.242	10.571	4.381	0.019	0.356
Schooling (≤9 years)	−0.325	2.571	0.900	−0.012	-	-	-	-
Smoking (No)	−8.119	3.641	0.030	−0.211	-	-	-	-
Race/Color (White/Yellow)	−5.777	3.115	0.069	−0.169	-	-	-	-
Age (Years)	-	-	-	-	0.178	0.103	0.088	0.196
Sex (Male)	-	-	-	-	−4.579	3.482	0.194	−0.159
Monthly income (≥260 US$)	-	-	-	-	7.394	3.981	0.068	0.229
Constant	58.225	5.337	<0.001	-	30.383	9.276	0.002	-

^1^—Regression coefficient b; ^2^—Standardized coefficient Beta.

## Data Availability

The data are available upon reasonable request to the corresponding author.

## Abbreviations

The following abbreviations are used in this manuscript:

HRQoL	Health-Related Quality of Life
PCS	Physical Component Summary
MSC	Mental Component Summary
RAND-36	RAND 36-Item Health Survey
MDT	Multidrug therapy
DPD	Degree of Physical Disability
